# Development and validation of a novel risk model for predicting atrial fibrillation recurrence risk among paroxysmal atrial fibrillation patients after the first catheter ablation

**DOI:** 10.3389/fcvm.2022.1042573

**Published:** 2022-12-02

**Authors:** Guangling Li, Xiaomei Wang, Jing-jing Han, Xueya Guo

**Affiliations:** ^1^Lanzhou University Second Hospital, The Second Clinical Medical College of Lanzhou University, Lanzhou, China; ^2^Department of Cardiology, Lanzhou University Second Hospital, Lanzhou University, Lanzhou, China

**Keywords:** atrial fibrillation, radiofrequency catheter ablation, atrial fibrillation recurrence, prediction model, recurrence risk

## Abstract

**Aims:**

Several models have been developed to predict the risk of atrial fibrillation (AF) recurrence after radiofrequency catheter ablation (RFCA). However, these models are of poor quality from the start. We, therefore, aimed to develop and validate a predictive model for post-operative recurrence of AF.

**Materials and methods:**

In a study including 433 patients undergoing the first circumferential pulmonary vein isolation (CPVI) procedure, independent predictors of AF recurrence were retrospectively identified. Using the Cox regression of designated variables, a risk model was developed in a random sample of 70% of the patients (development cohort) and validated in the remaining (validation cohort) 30%. The accuracy and discriminative power of the predictive models were evaluated in both cohorts.

**Results:**

During the established 12 months follow-up, 134 patients (31%) recurred. Six variables were identified in the model including age, coronary artery disease (CAD), heart failure (HF), hypertension, transient ischemic attack (TIA) or cerebrovascular accident (CVA), and left atrial diameter (LAD). The model showed good discriminative power in the development cohort, with an AUC of 0.77 (95% confidence interval [CI], 0.69–0.86). Furthermore, the model shows good agreement between actual and predicted probabilities in the calibration curve. The above results were confirmed in the validation cohort. Meanwhile, decision curve analysis (DCA) for this model also demonstrates the advantages of clinical application.

**Conclusion:**

A simple risk model to predict AF recurrence after ablation was developed and validated, showing good discriminative power and calibration.

## Introduction

Atrial fibrillation (AF) is the most common cardiac arrhythmia in clinical practice. As of 2019, there were approximately 59.7 million cases of AF (including atrial flutter) worldwide (1). AF can lead to complications such as heart failure (HF) and stroke, increasing mortality and disability (2). Therefore, rhythm control of AF has become essential to treating and preventing complications. Many clinical studies have confirmed the efficacy and safety of radiofrequency catheter ablation (RFCA) for AF, which is significantly better than drug therapy in maintaining sinus rhythm and can significantly improve symptoms and quality of life of patients (3–5).

Unfortunately, post-operative recurrence of catheter ablation (CA) of AF is relatively common. The recurrence rate of AF after CA based on circumferential pulmonary vein isolation (CPVI) is between 20 and 45% (6, 7). Such high recurrence rates may counteract the benefits of CA, so identifying patients at high risk of recurrence after CA appears to increase operative success while reducing unnecessary CA procedures and costs. Therefore, there is an increasing clinical need to identify the individual risk of AF recurrence after CA.

Existing studies have shown that many risk factors are associated with the development of AF, including obesity, age, hypertension, and HF (8, 9). However, risk factors associated with post-operative recurrence of AF are not well established. The main predictors of AF recurrence after CA include age, duration of AF, left atrial diameter (LAD), atrial substrate (requires MRI assessment), and renal function (10–13). Therefore, it is necessary to combine several indicators to generate a clinical individual risk prediction model for AF recurrence after the CA.

In previous studies, 12 models have been developed to predict AF recurrence, but their performance was disappointing (14). During model development, only two studies (17%) (15, 16) correctly assigned predictor weights based on regression coefficients, while the remaining 83% had no relevant information or used incorrect methods (such as simply assigning one point per variable). Furthermore, 92% of studies did not have validation, which could lead to overfitting of the model and thus overestimate the performance of the model. Meanwhile, in all relevant studies, no discrimination or calibration measures were reported in 30% of the analyses (14). We, therefore, developed and validated a predictive model to identify the individual risk of AF recurrence after CA. In previous studies by our team, the superiority of the C2HEST score (our previous research) for predicting post-operative recurrence in patients with AF has been demonstrated (17). Therefore, we would also further compare the two models.

## Materials and methods

### Patient population and study design

This study was a retrospective cohort study. We consecutively included all patients who underwent RFCA of AF in Lanzhou University Second Hospital from April 2018 to August 2021. To our best knowledge, no previous studies have established a prediction model specific to paroxysmal atrial fibrillation (PAF). However, recent studies have confirmed that RFCA is safe and effective as the preferred treatment for symptomatic PAF, which provides a support for RFCA as the first-line treatment of PAF, and the recurrence rate of PAF patients after CA also lower (5, 18, 19). Consequently, only patients with PAF were included in this study. For the study, the patient inclusion criteria were as follows: (1) patients were 18 years old or greater; (2) The patient was diagnosed with PAF by 12-lead ECG or 24 h Holter ECG; (3) The patient met the indications and underwent first RFCA of AF. The exclusion criteria for patients were as follows: (1) patient has a medical history of valvular heart disease (such as valvular stenosis, valvular insufficiency); (2) The patient has previously received AF ablation (including RFCA, cryoballoon ablation, surgical maze III and IV procedure); (3) Acute liver and kidney insufficiency or other reasons cause the inability to complete the procedure. (4) No detailed information describes the patient’s procedure (e.g., whether it was based on CVPI or linear ablation was added); (5) Patients were followed up for less than 6 months after the procedure. Finally, 433 patients were included in the final analysis and divided into training and validation sets according to a ratio of 7–3. PAF was defined as discontinuation within 7 days of onset, either automatically or after intervention (20). For risk factors (underlying disease) involved in the recurrence of AF after RFCA, the International Classification of Diseases, Ninth Revision, Clinical Modification (ICD-9-CM) was used as the diagnostic criteria.

The study was approved by the Ethics Committee of the Second Hospital of Lanzhou University. All patients had signed the informed consent before RFCA. In addition, the study was approved for visa-free clinical trial informed consent. The study observed the 1964 Declaration of Helsinki and its later amendments.

### Radiofrequency ablation strategy

Preoperative 12-lead ECG and 24 h Holter monitoring were performed to evaluate the patient’s heart rhythm. Simultaneously, transthoracic and transoesophageal echocardiography were performed to evaluate the patient’s cardiac structure and to exclude left atrial thrombus. Besides, pulmonary vein CT and three-dimensional cardiac imaging were used to evaluate pulmonary vein and left atrial structure. Patients taking antiarrhythmic drugs (AADs) mainly including amiodarone and propafenone before RFCA should discontinue the drug for at least five half-lives.

All included patients with AF underwent ablation procedures based on CPVI. Intravenous fentanyl 1 ug/kg was administered at the beginning of the RFCA, followed by continuous fentanyl 1 ug/(kg/h) infusion. A decapolar catheter was placed from the patient’s right internal jugular vein to the coronary sinus. Then, the right femoral vein was punctured, and a septal sheath and a septal needle were inserted. If the right internal jugular vein fails or is anatomically abnormal, the right femoral vein may be selected. After successfully puncturing the interatrial septum, a 3.5 mm irrigated-tip ablation catheter was introduced, modeled under the guidance of CARTO3, for CPVI ablation with 30–40 W of energy and a set maximum temperature of 43^°^C. During ablation of the posterior wall, the RFCA power was reduced to 25 W to reduce the risk of damaging surrounding structures. Whether to perform additional ablation (such as linear ablation or complex fractionated atrial electrogram ablation) was up to the electrophysiologist. A fairly conservative strategy for additional ablations was followed. Cavotricuspid isthmus ablation was performed in patients with documented typical atrial flutter. The failure of pulmonary vein pacing to outward conduction can confirm efferent block, but it is necessary to avoid far-field capture of adjacent atrial tissue which lead to misjudgment. Observe 30 min after CPVI and verify the bidirectional block between the left atrium and pulmonary veins.

The patient was free of bleeding within 5 h after CA and resumed oral anticoagulant use. Oral anticoagulants were maintained for 6 months, and after 6 months, the drug was discontinued or continued according to the CHA2DS2-VASc criteria. All patients with AF were treated with amiodarone (amiodarone 200 mg orally, three times a day for 4 weeks, followed by 200 mg orally, once a day for maintenance therapy) or propafenone (propafenone 300 mg orally, three times a day for maintenance therapy), and AADs were discontinued after 3 months.

### Study endpoint and patient follow-up

The study endpoint was a late recurrence of AF, which could be symptomatic or asymptomatic, defined as any atrial arrhythmia (including atrial tachycardia, atrial flutter, and AF) lasting more than 30 s between 3 and 12 months after RFCA. There were at least four outpatient follow-ups (3rd, 6th, 9th, and 12th months) after ablation, and 12-lead ECG and 24 h Holter monitoring were required for each follow-up. If they experienced symptoms of AF recurrence after the blank period to the end of the follow-up period, an ECG or electrocardiographic event recording should be performed immediately. When the patients did not follow up as planned, the patients were followed up by telephone. It should be emphasized that patients who still could not stop AADs after blank period were considered to have a AF recurrence.

### Statistical analysis

To avoid overfitting during model building, at least 10 events per variable were performed (21). Considering that the type of data missing was Missing At Random and the missing data was less than 20%, multiple imputation was conducted based on the predictive mean matching method (22). A total of five-fold multiple imputation were performed, and the maximum number of iterations for each imputation was 50, and the results were combined for analysis. The study population was split into development and validation cohorts by randomizing in a ratio of 7–3.

Continuous variables were expressed as mean ± standard deviation (SD) or median [interquartile range (IQR)], and statistical differences were estimated using the independent samples *t*-test or the Mann–Whitney U test. Categorical variables were presented as frequencies (percentages), and differences between groups were compared using the χ^2^ test or Fisher’s exact test. The statistical significance level was set at a two-sided *P* < 0.05. Follow-up time was calculated from data received at the first RFCA until data reached the primary study endpoint. Univariate proportional-hazards Cox regression was used to identify predictors of post-operative AF recurrence in the development cohorts. Variables were evaluated, including age, gender, chronic obstructive pulmonary disease (COPD), coronary artery disease (CAD), hypertension, HF, hyperthyroidism, transient ischemic attack (TIA) or cerebrovascular accident (CVA), hyperlipidemia, hyperuricemia, diabetes, obstructive sleep apnea hypopnea syndrome (OSAHS), chronic kidney disease (CKD), LAD, and left ventricular ejection fraction (LVEF). It should be noted that HF is divided into heart failure with reduced ejection fraction (HFrEF), heart failure with mid-range ejection fraction (HFmEF), and heart failure with preserved ejection fraction (HFpEF), so LVEF was not included in univariate proportional-hazards Cox regression analysis. In univariate analysis, variables with *p* < 0.05 were included in the multivariate Cox regression model. Then stepwise regression is then performed based on the Akaike information criterion (AIC) to obtain the optimal model while preventing overfitting. 12 months survival nomogram was plotted based on the final multivariate Cox regression model.

In development cohorts, the area under the curve (AUC) was used to evaluate the discriminative power of the survival nomogram. AUC > 0.7 indicates that the model has a high discriminative ability. A calibration curve was performed to assess the accuracy of the survival nomogram. The predictive ability of the newly built model and the C2HEST model is then compared using the Net Reclassification Index (NRI) and Integrated Discrimination Improvement (IDI). Besides, decision curve analysis (DCA) curves were drawn to assess the clinical benefit of the prognostic model (23). Nomogram was derived and validated against checklists in the Transparent Reporting of a Multivariable Prediction Model for Individual Prognosis or Diagnosis (TRIPOD) guideline (24). Further, we use validation cohorts to perform an internal validation. The AUC curve, calibration curve, and DCA were plotted again in the validation cohort, and then NRI and IDI were calculated to compare the two models.

## Results

### Characteristics of the study population

**FIGURE 1:**
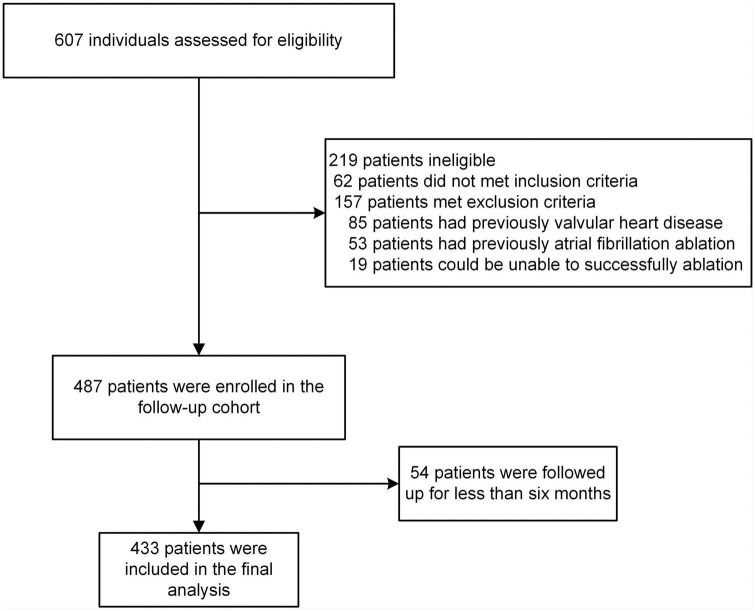
Patient was screened and followed up.

A total of 706 patients were included in the screening, and 433 were involved in the final analysis (Figure 1). Baseline characteristics of the study population were presented in Table 1. Overall, the development and validation cohort were balanced. In the development cohort, the mean age of patients was 60.30 (±10.50) years, and 190 (62.3%) were male. In the validation cohort, the mean age of patients was 59.11 (±9.36) years, and 78 (60.9%) were male. During the follow-up period, the median survival time (the time from the post-operative follow-up to the first recurrence) of patients in the development cohort was 12.00 [IQR, 35.00, 45.00] months, and in the validation cohort was 12.00 [IQR, 10.00, 12.00], there was no statistically significant difference between the two groups (*P* < 0.05). In addition, during the follow-up period, the number of patients who relapsed in development cohorts was 96 (31.5%), and in the validation cohort was 38 (29.7%), with no statistically significant difference between the two groups (*p* > 0.05).

**TABLE 1:** Patients characteristics of the development and validation cohorts.

Variables	Development cohorts	Validation cohorts	*p*
	(*N* = 305)	(*N* = 128)	
Age (mean [SD])	60.30 (10.50)	59.11 (9.36)	0.27
Gender (%)	190 (62.3)	78 (60.9)	0.88
**Previous medical history**			
COPD (%)	14 (4.6)	6 (4.7)	1.00
CAD (%)	72 (23.6)	28 (21.9)	0.79
Hypertension (%)			0.70
Grade hypertension 1	46 (15.1)	25 (19.5)	
Grade hypertension 2	36 (11.8)	13 (10.2)	
Grade hypertension 3	50 (16.4)	21 (16.4)	
HF (%)			0.73
HFrEF	8 (2.6)	3 (2.3)	
HFmrEF	14 (4.6)	9 (7.0)	
HFpEF	72 (23.6)	27 (21.1)	
Hyperthyroidism (%)	17 (5.6)	7 (5.5)	1.00
TIAorCVA (%)	35 (11.5)	15 (11.7)	1.00
Hyperlipidemia (%)	92 (30.2)	36 (28.1)	0.76
Hyperuricemia (%)	33 (10.8)	21 (16.4)	0.15
Diabetes (%)	45 (14.8)	13 (10.2)	0.26
OSAHS (%)	59 (19.3)	19 (14.8)	0.33
CKD (%)	57 (18.7)	20 (15.6)	0.53
**Imaging**			
LAD (median [IQR])	40.00 [35.00, 45.00]	38.95 [35.00, 44.70]	0.57
LVEF (median [IQR])	61.00 [55.00, 65.00]	64.00 [57.75, 66.00]	0.05
E/E′ (median [IQR])	12.00 [8.00, 16.00]	12.00 [8.00, 14.25]	0.46
AADs			0.70
Amiodarone	244 (80.0)	101 (78.9)	
Propafenone	37 (12.1)	14 (10.9)	
Other AADs	24 (7.89)	13 (10.2)	
Survival time (median [IQR])	12.00 [9.00, 12.00]	12.00 [10.00, 12.00]	0.94
AF recurrence (%)	96 (31.5)	38 (29.7)	0.80

Values are presented as *n* (%), mean (SD), or median (IQR). COPD, chronic obstructive pulmonary disease; CAD, coronary artery disease; HFrEF, heart failure with reduced ejection fraction; HFmrEF, heart failure with mid-range ejection fraction; HFpEF, heart failure with preserved ejection fraction; TIA, transient ischemic attack; CVA, cerebrovascular accident; OSAHS, obstructive sleep apnea hypopnea syndrome; CKD, chronic kidney disease; LAD, left atrial diameter; LVEF, left ventricular ejection fraction.

E/E’ represents the ratio of the peak flow velocity of the mitral valve in early diastole and the peak flow velocity of the mitral annulus in late diastole; AADs, antiarrhythmic drugs.

### Development cohort

**FIGURE 2:**
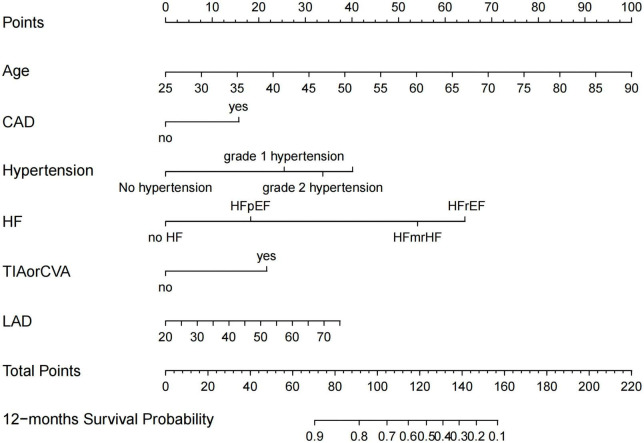
Nomogram for predicting atrial fibrillation (AF)-free survival probability between 3 and 12 months after radiofrequency catheter ablation (RFCA). CAD, coronary artery disease; HFrEF, heart failure with reduced ejection fraction; HFmrEF, heart failure with mid-range ejection fraction; HFpEF, heart failure with preserved ejection fraction; TIA, transient ischemic attack; CVA, cerebrovascular accident; LAD, left atrial diameter.

**FIGURE 3:**
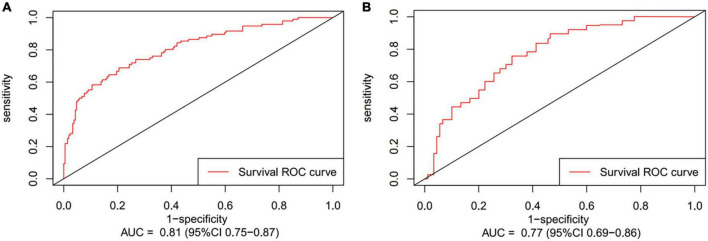
The receiver operating characteristic curves (ROC) of post-operative 12 months follow-up present area under the curve (AUC) in development cohort **(A)** and validation cohort **(B)**.

**FIGURE 4:**
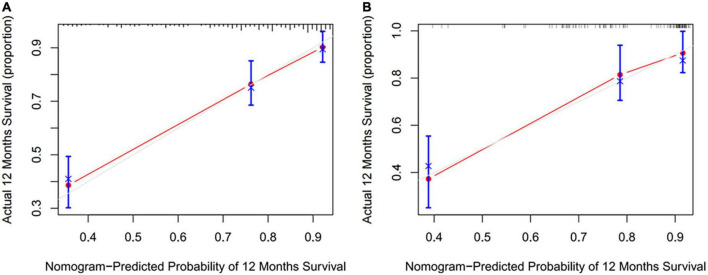
The calibration curves were predicted by nomogram in development cohort **(A)** and validation cohort **(B)**.

Preoperative variables were performed to univariate Cox regression analysis. The analysis identified ten variables (Age, CAD, COPD, hypertension, HF, TIAorCVA, hyperlipidemie, diabetes, OSAHS, LAD) highly associated with AF recurrence between 3 and 12 months after RFCA. Further, these 10 variables were included in a multivariate Cox regression analysis, and six independent variables (including Age, CAD, hypertension, HF, TIAorCVA, and LAD) were obtained to predict the AF recurrence after RFCA (Table 2). The results of multivariate Cox regression analysis were used to generate a nomogram to predict the risk of AF recurrence between 3 and 12 months after RFCA (Figure 2). The receiver operating characteristic curves (ROC) of post-operative 12 months follow-up indicated that the model had high predictive power, with an AUC of 0.81 (95% confidence interval [CI], 0.75–0.87) (Figure 3A). The calibration curve suggested good consistency between predicted and actual probabilities, demonstrating a good fit (Figure 4A).

**TABLE 2:** Multivariate Cox regression of atrial fibrillation (AF) recurrence risk between 3 and 12 months after radiofrequency catheter ablation (RFCA) for patients with paroxysmal atrial fibrillation (PAF).

Variables	aHR	Lower 95%	Upper 95%	*P*
Age	1.06	1.03	1.08	<0.001
CAD	1.75	1.13	2.72	0.013
**Hypertension**				
Grade 1	2.48	1.33	4.63	0.004
Grade 2	3.34	1.86	6.00	<0.001
Grade 3	4.19	2.49	7.06	<0.001
**HF**				
HFrEF	9.91	4.23	23.26	<0.001
HFmrEF	6.90	3.04	15.67	<0.001
HFpEF	1.92	1.19	3.09	0.007
TIAorCVA	2.17	1.26	3.73	0.005
LAD	1.02	1.00	1.05	0.078

aHR, adjusted hazard ratio; CAD, coronary artery disease; HFrEF, heart failure with reduced ejection fraction; HFmrEF, heart failure with mid-range ejection fraction; HFpEF, heart failure with preserved ejection fraction; TIA, transient ischemic attack; CVA, cerebrovascular accident; LAD, left atrial diameter.

### Validation cohort

The model derived from development cohort was tested using internal validation. Similarly, in the validation cohort, the model also showed good predictive ability with an AUC of 0.77 (95% CI, 0.69–0.86) (Figure 3B). Simultaneously, it also shows a high degree of consistency in the internally verified calibration curve (Figure 4B), where the nomogram-predicted probability of 12 months survival (*x*-axis) matched the actual 12 months survival probability (*y*-axis).

### Clinical application

**FIGURE 5:**
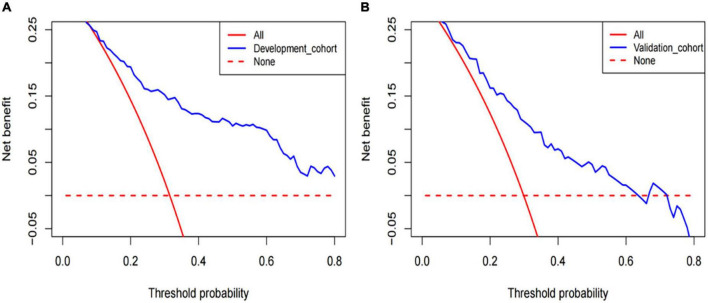
The decision curve analysis (DCA) was performed in development cohorts **(A)** and validation cohorts **(B)**.

The DCA of the model in development cohort (Figure 5A) and validation cohort (Figure 5B) suggests better application ability. It can be observed from the decision curve that the model provides a clear net benefit relative to “all individuals with AF recurrence” or “no individuals with AF recurrence.”

### Comparing with C2HEST model

In our previous study, we have confirmed that the C2HEST score, including CAD and COPD (each gets one point), H: hypertension (one point), E: elderly (age ≥ 75 years, two points), S: systolic HF (two points), and T: thyroid disease (hyperthyroidism, one point), can be used to predict the AF recurrence after RFCA, and it has excellent discriminative power. In the development cohort, when the C2HEST score is used as the standard model, and the model established by development cohort is used as the new model, the calculated NRI (Table 3) was 0.085 (95% CI, −0.090–0.173), and the two models do not show a statistical difference in the accuracy of predictive ability. Further, the calculated IDI was 0.015(*P* = 0.096), indicating that the new model did not significantly improve its predictive power compared to the standard model. In validation cohorts, similar results were observed. NRI and IDI were −0.089 (95% CI, −0.169–0.160) and 0.002 (*P* = 0.418), respectively, with no statistical significance.

**TABLE 3:** The calculated net reclassification index (NRI) in the development cohort and validation cohort.

Group	Estimate	Lower (95% CI)	Upper (95% CI)
**Development cohort**			
NRI	0.085	−0.059	0.175
NRI+	0.042	−0.063	0.106
NRI-	0.043	−0.036	0.096
**Validation cohort**			
NRI	−0.089	−0.169	0.160
NRI+	0	−0.121	0.160
NRI-	−0.089	−0.121	0.097

NRI, net reclassification index; CI, confidence interval. NRI+ means to calculate NRI in the AF recurrence patients. NRI- means to calculate NRI in the without AF recurrence patients.

## Discussion

### Main findings and significance of the study

This study developed a new model to predict the risk of late recurrence of AF after RFCA in development cohort. The model had good discriminative power with an AUC of 0.81 (95% CI, 0.75–0.87), and the calibration curve also suggested good agreement between predicted and actual probabilities. After further clinical DCA, it was found that predicting AF recurrence based on this model could lead to a net clinical benefit. In validation cohort, the performance of the model is similar to that in development cohort, further indicating that the model is stable while reducing the risk of overfitting.

A recent meta-analysis summarized 33 studies on 13 models for predicting post-operative AF recurrence (14). Unfortunately, no single model consistently has poor or good discriminative power across related studies and is highly variable across studies (25–32). Only two of these studies were evaluated for model calibration. Furthermore, none of the studies were evaluated for internal validation in the model established, which may lead to the overfitting of the model. Therefore, there is the fact that models were often poorly developed in the first place. To address the above issues, we conducted this study. To the best of our knowledge, this is the first study to conduct internal validation early in model development. Simultaneously, this is also the first predictive model specifically for post-operative recurrence of PAF patients. Most of the previous studies included various types of AF patients. Although this increases the extrapolation of the model, it also increases the heterogeneity within studies. A fact that we should not ignore is that PAF patients are the main body of AF patients and also the primary population for RFCA.

### New model as a predictor for atrial fibrillation recurrences

In our newly developed model, the following risk factors were included: age, CAD, hypertension, HF, TIA or CVA, and LAD. Previous studies have shown that aging is a risk factor for AF, and the incidence of AF has been increasing with years. Elderly patients are often accompanied by various chronic diseases, the metabolic clearance capacity of AADs is reduced, and drug-related arrhythmias are more likely to occur. Besides, some studies suggest that the short-term success rate of CA for AF in elderly patients with indications (>75 years old) is comparable to that in younger patients, and there is no significant difference in the incidence of complications (25). The above evidence suggests that elderly patients with AF may benefit from RFCA. However, it should be noted that increasing age may be accompanied by an aggravation of myocardial fibrosis, and myocardial fibrosis is an independent risk factor for recurrence after AF ablation. In recent studies, aging has also been shown to be a risk factor for AF recurrence after RFCA (17, 26–28). Obviously, RFCA for elderly patients with AF is a trade-off procedure. For patients with CAD, it can cause chronic ischemia of the myocardium, and this process will produce myocardial fibrosis, which is the substrate of AF, promoting the formation of reentry and maintaining the onset of AF (29). At the same time, this risk factor has also been confirmed in other studies predicting AF recurrence (30–32).

Epidemiological studies suggest that hypertension is the most crucial risk factor in patients with AF (33). If blood pressure is not well controlled, the risk of developing AF in hypertensive patients is significantly increased (34). The mechanism may be related to increased left atrial pressure, atrial fibrosis, and inflammatory cell infiltration (34). Hypertension also predicts the risk of AF recurrence after RFCA, but there is currently insufficient evidence that aggressive blood pressure control improves ablation success (35). In patients with decreased LVEF or left ventricular hypertrophy, angiotensin converting enzyme inhibitors and angiotensin receptor blockers may reduce the risk of post-operative AF recurrence (36). Besides, in our study, it was found that patients with higher hypertension also had a higher risk of post-operative AF recurrence, which was confirmed in previous studies (17). In our study, HF increased the risk of post-operative AF recurrence. This result has also been confirmed in other studies (37, 38). However, some studies have confirmed that the success rate of RFCA in HF with AF patients is not significantly different from that in AF without HF patients (39). After further comparison, the most likely reason is the difference in the included population because the above study is for hypertrophic obstructive cardiomyopathy, which obviously does not represent the majority of patients with HF. The CASTLE-AF study showed that compared with medical therapy, RFCA in patients with HF and AF can reduce all-cause mortality and hospitalization due to worsening HF (40). Therefore, in clinical patients with AF and HF, RFCA should be the preferred treatment option.

TIA or CVA often exists as a complication of AF. In addition, they are also risk factors for predicting the risk of stroke in patients with AF. There are few previous studies on TIA or CVA leading to post-operative AF recurrence. As far as we know, several studies have verified TIA or CVA as a risk factor for post-operative AF recurrence (17, 41). Furthermore, increased LAD leads to an increased chance of reentry, as well as structural remodeling of the atrium, which contributes to the development of AF. The present study further demonstrates that increased LAD is also significantly associated with post-operative AF recurrence (30, 31, 42) and that LAD is the most common risk factor in all predictive models.

### New model and C2HEST model

The C2HEST model was initially used to evaluate the risk of AF in individuals. But many of its risk factors are related to the recurrence of AF, so it was innovatively used by us to evaluate the recurrence of AF after RFCA and was verified. However, the model was not initially used to evaluate the post-operative recurrence of AF, so there was a particular bias from the beginning. In addition, we also found that the population with paroxysmal AF has not been specifically studied in previous studies, but this is the main body of AF patients and the main population for the long-term benefit of RFCA (the C2HEST model also includes paroxysmal AF and persistent AF patients). Finally, combined with previous post-operative recurrence prediction models for AF, the establishment is inferior (none of the models have been reasonably internally validated at the beginning of establishment). To address the above three problems, we conducted this study. Even though the model we built did not show better predictive ability than the C2HEST model, it is evident that the two models are different (including the purpose of building the model and the study subjects), so the clinical significance of the model cannot be denied.

### To help clinical decision

In our new model, all risk factors are available before RFCA, which means it can be used for preoperative guidance. The risk of recurrence in AF patients can be thoroughly evaluated before RFCA. That is to say, the risk of AF recurrence can be predicted based on the nomogram. Therefore, we can hypothesize that patients with a predicted recurrence risk of less than lower limit of recurrence rate in our center are the most suitable population for CA. For patients with a recurrence risk higher than upper limit of recurrence, CA is generally not recommended to avoid an unnecessary procedure. It should be noted that post-operative AF recurrence is definitely related to the type of AF and screening methods for recurrence (43, 44). Due to the fact that we screen for AF more frequently and more strictly defined, the recurrence rate will be higher than reported in the literature. In our study, it has been clarified that the research subject is PAF patients, and a clear definition of the screening program has also been given, so its scope of application is clear. Furthermore, it should be emphasized that clinical decision-making is a complex process that requires multiple pieces of evidence, so this model only provides more reference for clinicians. Obviously, whether its applicable subject can be further extrapolated and how its benefits will require further clinical research.

## Study limitations

This study has several limitations. First, the study was single-center and could not be extrapolated to other centers. In addition, this study employed RFCA based on CPVI and, therefore, could not be easily generalized to other operation treatments. Second, the AF recurrence may be asymptomatic, so it is vital to strengthen the monitoring of heart rhythm. However, in our study, an implantable ECG event recorder was not used, so the AF recurrence rate may be underestimated to some extent. Third, and most importantly, we did not perform an external validation of the model, which could lead to an under-evaluation of the model. Obviously, to resolve the above problems, a multicenter, large sample study is needed to monitor patients after RFCA continuously.

## Conclusion

A new model was developed to predict the 12 months risk of AF recurrence after RFCA. The variables included in the model included age, CAD, hypertension, HF, TIA or CVA, and LAD. The model presents good discriminative power and calibration. In addition, the model demonstrated a net clinical benefit in DCA. Further, the above conclusion are confirmed in the verification cohorts.

## Data availability statement

The original contributions presented in this study are included in the article/supplementary material, further inquiries can be directed to the corresponding author.

## Ethics statement

The studies involving human participants were reviewed and approved by the Ethics Committee of the Second Hospital of Lanzhou University. The ethics committee waived the requirement of written informed consent for participation. Written informed consent was not obtained from the individual(s) for the publication of any potentially identifiable images or data included in this article.

## Author contributions

GL completed the follow-up of patients, the data analysis, and wrote the manuscript. XG guided this clinical trial. J-JH and XW completed the manuscript revision. All authors listed have made a substantial, direct, and intellectual contribution to the work, and approved it for publication.
